# Shelterbelt Poplar Forests Induced Soil Changes in Deep Soil Profiles and Climates Contributed Their Inter-site Variations in Dryland Regions, Northeastern China

**DOI:** 10.3389/fpls.2019.00220

**Published:** 2019-03-05

**Authors:** Yan Wu, Qiong Wang, Huimei Wang, Wenjie Wang, Shijie Han

**Affiliations:** ^1^Department of Biological Engineering, Da Qing Normal University, Daqing, China; ^2^Department of Life Science, Henan University, Kaifeng, China; ^3^Key Laboratory of Forest Plant Ecology, Northeast Forestry University, Harbin, China

**Keywords:** poplar shelterbelt, farmlands, soil properties change, deep-layer soil, analysis of causes

## Abstract

The influence of shelterbelt afforestation on soils in different-depth profiles and possible interaction with climatic conditions is important for evaluating ecological effects of large-scale afforestation programs. In the Songnen Plain, northeastern China, 720 soil samples were collected from five different soil layers (0–20, 20–40, 40–60, 60–80, and 80–100 cm) in shelterbelt poplar forests and neighboring farmlands. Soil physiochemical properties [pH, electrical conductivity (EC), soil porosity, soil moisture and bulk density], soil carbon and nutrients [soil organic carbon (SOC), N, alkaline-hydrolyzed N, P, available P, K and available K], forest characteristics [tree height, diameter at breast height (DBH), and density], climatic conditions [mean annual temperature (MAT), mean annual precipitation (MAP), and aridity index (ARID)], and soil texture (percentage of silt, clay, and sand) were measured. We found that the effects of shelterbelt afforestation on bulk density, porosity, available K, and total P were observed up to 100 cm deep; while the changes in available K and P were several-fold higher in the 0–20 cm soil layer than that in deeper layers (*p* < 0.05). For other parameters (soil pH and EC), shelterbelt-influences were mainly observed in surface soils, e.g., EC was 14.7% lower in shelterbelt plantations than that in farmlands in the 0–20 cm layer, about 2.5–3.5-fold higher than 60–100 cm soil inclusion. For soil moisture, shelterbelt afforestation decreased soil water by 7.3–8.7% in deep soils (*p* < 0.05), while no significant change was in 0–20 cm soil. For SOC and N, no significant differences between shelterbelt and farmlands were found in all five-depth soil profiles. Large inter-site variations were found for all shelterbelt-induced soil changes (*p* < 0.05) except for total K in the 0–20 cm layer. MAT and silt content provided the greatest explanation powers for inter-site variations in shelterbelt-induced soil properties changes. However, in deeper soils, water (ARID and MAP) explained more of the variation than that in surface soils. Therefore, shelterbelt afforestation in northeastern China could affect aspects of soil properties down to 100 cm deep, with inter-site variations mainly controlled by climate and soil texture, and greater contribution from water characteristics in deeper soils.

## Introduction

Globally, ecological shelterbelt engineering projects, such as the Great Plains Shelterbelt Project (Roosevelt Engineering) in the USA, the Great Plan for the Transformation of Nature in the former Soviet Union, forestry and water conservation projects in Japan, the Green Dam Engineering Project in the five countries of North Africa, and the Three-North Shelterbelt Program in China, have increased the scientific study of shelterbelt forests (Zhang et al., 2016). There are numerous forest plantations worldwide, many of which were planted in degraded or abandoned farmlands and are used as agricultural protection forests or bioenergy forests in China (Wang G. Y. et al., 2018; Zhang et al., 2018) and worldwide (Deniz and Paletto, 2018; Jha, 2018). The area of shelterbelt forests used for protecting soil and water increased to 330 million ha globally by 2010, accounting for 8% of all forest areas. The largest proportion of shelterbelt forests is in Asia (26%), 33% of which are in East Asia, and China's shelterbelt forests account for most of that area (60 million ha of the total 83 million ha) (Obschatko et al., 2010). There are approximately 6.67 million ha of poplar plantations that are widely distributed in China. The large shelterbelt forest area in China makes it a good example for studying the ecological functions of shelterbelt forests, and underground soil changes are an important issue to fully understand the functions of forests (Zhu, 2013; Wang et al., 2015, 2017b; Wu and Wang, 2016; Wang Q. et al., 2017; Zhong et al., 2017; Nan et al., 2018).

Black soils in northeastern China are mainly located in the Songnen Plain and Sanjiang Plain, which contain one of the three global black soil belts, and over 45% of the total grain output in northeastern China is produced in this region (Wang et al., 2009b). Although the black soils in northeastern China contain abundant soil organic matter and have high fertility compared with other soils (Cas, 1980; Hljtr, 1992), excessive historical reclamation has led to sharp decreases in soil fertility since the establishment of the People's Republic of China in 1949 (Wang et al., 1996) and nearly half of the nitrogen and soil organic matter has been lost from the black soils in northeastern China (Ding and Liu, 1980; Wang, 2002; Wang et al., 2011b). Several studies have shown that afforestation in cultivated farmland soils induced changes in most soil properties and soil fertility, contributing to soil improvement in different cases (Li and Cui, 2000; Wang Q. et al., 2014; Wang et al., 2017a). Shelterbelts of different ages and tree species could effectively reduce nitrate nitrogen by 22–60% (Jaskulska and Jaskulska, 2017), and also regulate soil physiochemical properties, fertility, and carbon sequestration (Wang et al., 2017a). In addition, soil physical properties could be altered from afforestation practices, including increases in soil bulk density and decreases in total porosity, water retention, and ventilation capacity (Wang, 2002; Wang et al., 2011b, 2017a). However, other studies also found that fast-growing plantations, such as larch, poplar, or eucalyptus, require more soil nutrients, and water (Chen, 1998; Mendham et al., 2003; Merino et al., 2004; Zhang et al., 2004; Li Y. et al., 2018), which is possibly induced by deep soil changes in various soil properties (Wang H. M. et al., 2014; Wang W. J. et al., 2014). Most of these studies have been undertaken in surface soils < 40 cm deep with the assumption of neglectable changes in deep soils relative to surface soils. However, other studies have found that deep soils can sensitively react to land use changes (Fontaine et al., 2010; Rukshana et al., 2011), especially for tree species that have relatively longer roots compared to crops (Wang Q. et al., 2014; Wang S. et al., 2017).

Songnen Plain was named after the Songhua and Nenjiang Rivers running through this region. This plain has been recognized as the northern-most region of the Three-North Shelterbelt Program (Wu and Wang, 2016). Songnen Plain is about 18.28 million ha and locates in the transitional region between the semi-moist and semi-arid region, featured as saline-alkalinization and heavy farmland soil degradation (Li, 2000; Wang et al., 2011a) as well as natural forest degradation (Dai et al., 2018). Our previous study has shown that poplar shelterbelt afforestation in northeastern China slightly changed SOC sequestration and N nutrients in the surface (20 cm) soils, with sharp decreases in bulk density (Wu et al., 2018), with no consideration in deep soils (>20 cm). Moreover, glomalin-related soil carbon sequestration was higher in deep soils than that in surface soils, with more response to climatic changes in the farmlands of this region (Wang et al., 2017b). Most poplar roots concentrated in the 0–60 cm soils, and the influence of vegetation growth and microbial activities on soils may extend over the depth of the roots (Jobbágy and Jackson, 2000). Annual precipitation in Songnen Plain ranges from 300 to 500 mm, with a 2–3-fold higher annual evaporation (1,000–1,500 mm) (Li, 2000). This natural background, heavy pressure from farming and grazing, and fast saline-alkalinization in soil are important challenges for social development and livelihood in this region (Li, 2000; Wang et al., 2011a). The evaluation of shelterbelt afforestation on underground soils in this region must fully consider the variations in the widespread plain, and fully understanding of the underlying mechanisms needs more consideration on forest characteristics, climatic conditions including the aridity index (ARID), soil texture both at surface and deep soils (Wang W. J. et al., 2014; Wu et al., 2018).

In the present study, we alleged that deep soils at 100 cm depth should be included in the evaluation of various soil changes in poplar afforestation, and large inter-site variation in the shelterbelt forest-induced soil changes were related to local climatic differences, soil texture, and forest growth. We posed several research questions as follows: (1) Should deep soil layers be included in the evaluations of soil improvements from degraded farmlands to poplar forests and did these improvements differ in different soil parameters? (2) How great a difference among locations occurred in the shelterbelt-induced soil changes, and which factors of climatic condition, soil texture, and tree growth parameter were responsible for these variations? By evaluating the shelterbelt-induced soil changes in various properties in different soil layers, our data assisted the evaluation of underground soil changes in large scale shelterbelt programs, such as the Three-North Shelterbelt Program, particularly the quantification of the importance of deep soils for afforestation practices in degraded farmlands.

## Materials and Methods

### Study Sites and Sample Collection

**Figure 1:**
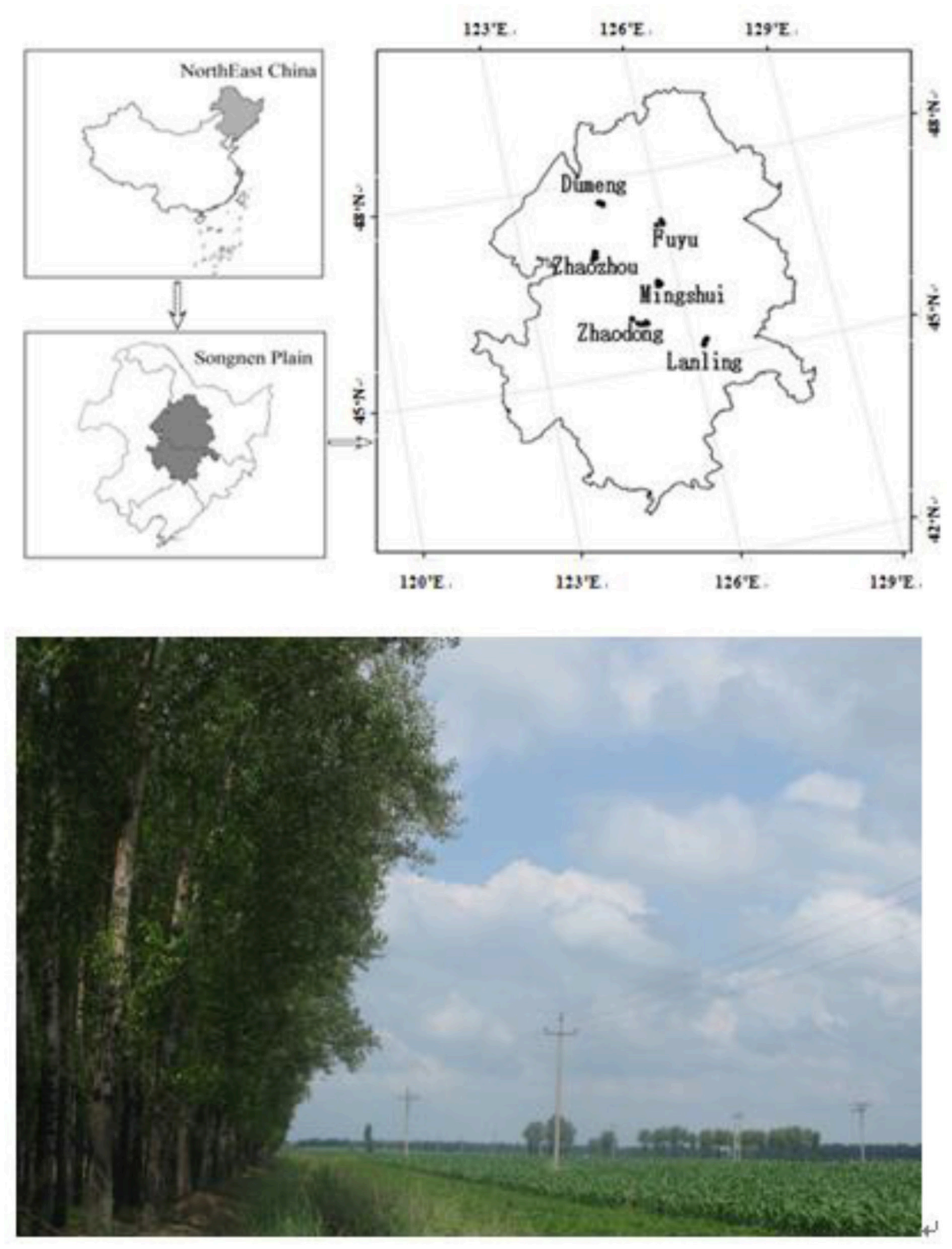
Six study sites in the Songnen Plain, Northeastern China, and a typical poplar shelterbelt-farmland paired site. Parts of this figures was adapted from our previous publication (Wu et al., 2018).

The Three-North Shelterbelt Program established tree plantations around farmlands in northern China, northwestern China, and northeastern China in 1978 (Zhu, 2013). The general design was to plant 4–10 rows of poplars around 500 × 500 m of farmland, and large areas of shelterbelts around farmlands are found everywhere throughout the Songnen Plain of northeastern China (Figure 1). Nowadays, the most-used poplar variety in northeast regions (young forests) is Yinzhong poplar (*Populus alba* × *Populus berolinensis*), while historically, the most-planted poplars were *Populus simonii, Populus* × *xiaohei*, and *Populus deltoides* × *P. canadensis*, etc. (Wu et al., 2018).

Six study sites (Dumeng, Fuyu, Lanling, Mingshui, Zhaodong, and Zhaozhou) distributed in the Songnen Plain in the middle of northeastern China were selected as study sites (Figure 1). The soil types in the study region are typical black soils, including Chernozem (Fuyu, Lanling), Phaeozem (Mingshui), Cambosols (Dumeng), and some degraded soil, such as Solonetz (Zhaozhou, Zhaodong). This region has a continental monsoon climate, with MAT of 2.9–4.4°C, MAP of 350–500 mm, and ARID of 0.4–0.7.

Soil samples were collected from 72 paired shelterbelt plantations and farmland plots in the six study sites. Five soil profiles were collected from each paired plot. After the exclusion of the A0 layer, we sampled 100 cm of soil from 0 to 20, 20 to 40, 40 to 60, 60 to 80, and 80 to 100 cm depths. Additionally, we obtained a composite sample by mixing five samples from the same soil layers from each of the five soil profiles. In total, 720 soil samples (6 regions × 2 (farmland and shelterbelt) × 12 sites/region × 5 depths/site = 720 samples) were collected.

### Determination of Soil Parameters

Sample preparation details and some of the soil parameters analysis (e.g., bulk density, soil moisture, SOC, total N, available N, total K, available K, total P, available P, and soil texture) have been described previously by Wu et al. (2018). Soil porosity was calculated by the following formula: soil porosity = (1−bulk density/specific gravity) × 100%. The pH of the soil solution (one-part soil to five-parts water) was measured with an acidity meter (Sartorius PT-21, Shanghai, China). Soil electrical conductivity (EC) was determined with an EC meter (DDS-307, Shanghai Precision Scientific Instruments Co., Ltd., Shanghai, China) (Bao, 2000). Soil carbon or nutrient storage were computed as:

Farmland soil carbon or nutrient storage = α_*f*_ × ρ_*f*_ × 0.2 × (1–*V*_*gravel*_)

Poplar soil carbon or nutrient storage = α_*p*_ × ρ_*p*_ × 0.2 × ρ_f_/ρ_p_ × (1–*V*_*gravel*_)

where, α_*f*_ and α_*p*_ are the concentrations of farmland and poplar SOC (g kg^−1^); ρ_*f*_ and ρ_*p*_ are farmland and poplar soil bulk densities (Mg m^−3^), respectively; 0.2 is the soil thickness (0.2 m); and *V*_*gravel*_ is the proportion of gravel. Details regarding the bulk density correction can be found in Wuest (2009) and Wu et al. (2018).

### Forest Characteristics, Soil Texture, and Climatic Data Collection

Poplar forest characteristics of tree density, tree height, and DBH were measured at each plot site. Regarding the distance between forest and farmland, 42% (30 plots) of plots were < 3.4 m from neighboring farmland, while 47% (34 plots) were 3.4 to 6.7 m away. To reduce the influence of roots on neighboring farmlands, ditches of about 2 m in width and 2 m in depth were excavated between shelterbelt and farmland by local farmers. Forest characteristics data of 72 plots can be found in Wu et al. (2018).

The soil texture was the relative amount of sand, silt, and clay in the bulk soil, measured using a rapid and simple method described by Kettler et al. (2001) and Wu et al. (2018).

MAT and MAP at the six sites were obtained from the meteorological scientific data sharing service network of China (http://cdc.cma.gov.cn/) and the ARID was computed as the MAP over the mean annual reference evapotranspiration (Huo et al., 2013).

### Data Analysis

Calculation of storage at different soils depth, such as 0–20 cm, 0–40 cm, 0–60 cm, 0–80 cm, and 1 m soil profiles, is a general rule for many previous studies for ease of comparison among studies (Wang et al., 2011b; Wei et al., 2014; Wang H. M. et al., 2017a; Deng et al., 2018). In this paper, in order to compare soil carbon or nutrient storage with other studies, we have re-grouped our data from 0 to 20, 20 to 40, 40 to 60, 60 to 80, and 80 to 100 cm into 0 to 20, 0 to 40, 0 to 60, 0 to 80, and 0 to 100 cm by combining the corresponding soil layer's measured data. For example, 0–20 and 20–40 cm were combined into one set of 0–40 cm by average of two data; Similarly, combining 0–40 and 40–60 cm into 0–60 cm, combining 0–60 and 60–80 cm into 0–80 cm, and combining 0–60 and 80–100 cm into 0–100 cm during dada analysis in order to evaluate the effects of afforestation on soil properties in five soil-depth profiles.

Multivariate analysis of variance (MANOVA) was used to determine the influence of land use type (shelterbelt forest and neighboring farmland), sampling location (Dumeng, Fuyu, Lanling, Mingshui, Zhaodong, and Zhaozhou), and their interaction on various soil parameters. The 19 parameters (soil bulk density, soil porosity, soil moisture, pH, EC, SOC concentration, total N concentration, alkaline hydrolyzed N concentration, total P concentration, available P concentration, total K concentration, available K concentration, SOC storage, total N storage, alkaline hydrolyzed storage, total P storage, available P storage, total K storage, and available K storage) were used as dependent variables.

A paired *t*-test was used to determine the difference in soil properties between shelterbelt plantations and farmlands at different soil depths, and the Duncan's test was used for multiple comparisons among different soil-depth profiles for all shelterbelt-induced soil changes. In the present study, the relative change [(forest—farmland)/farmland] of each soil parameter was treated as a dependent variable for the following analysis.

Redundancy analysis (RDA) was conducted to ordinate the complex associations between shelterbelt-induced variations in various soil properties and climatic conditions, soil texture, and forest characteristics (Canoco 5.0 software program). Ordination was performed in all five soil-depth profile, as we wanted to find the differences in different soil depths. Conditional term effects (excluding collinear effects among different dependent parameters) were derived from the RDA, and the possible factors contributing to the dependent variables (e.g., shelterbelt-forest-induced changes in soil properties as a whole). In conditional term effects, the significant factor with the highest explanation percentage showed the strongest contribution to the variation of soil properties. Details explaining the RDA ordination can be found in previous studies (Wang et al., 2017b, 2018b).

Stepwise regression analysis was used to explore the factors responsible for the poplar-induced changes in various soil properties in the five soil layers. Statistical significance was evaluated at *p* = 0.05, unless otherwise stated. Three groups of parameters, forest characteristics (tree height, DBH, and tree density), soil texture (sand, silt, and clay), and climatic conditions (MAT, MAP, and ARID), were tested as independent parameters. The more entering times, and more frequent parameter into the stepwise models indicate the stronger influence from these parameters for explaining the variations of soil properties from shelterbelt afforestation. By using this criteria, stepwise regression models were analyzed for simplifying the presentation of the data and facilitate data interpretation.

## Results

### Land Use Type and Sampling Location Affect All Soil Parameters: Manova Results

Table 1 showed the influences of soil use type, sampling location, and their interaction on the soil parameters at five depths. Significant land use effects (farmland and poplar forest) were observed in bulk density, porosity, and total P storage for all five depths. Significant differences between the two land uses on pH, EC, available K concentration, and available K storage were found in the 0–20 cm depth, whereas others, such as soil moisture, total K, total P, available P concentration, and available P storage were statistically different among the two land uses in the deeper soil layers (>20 cm).

**Table 1:** Shelterbelt plantation establishment, sampling regions, influences various soil parameters, and possible interacts at different depths.

**Dependent Variable**	**0–20 cm**			**0–40 cm**			**0–60 cm**			**0–80 cm**			**0–100 cm**		
	**T**	**L**	T ^*^L	**T**	**L**	T ^*^L	**T**	**L**	T ^*^L	**T**	**L**	T ^*^L	**T**	**L**	T ^*^L
**PHYSIOCHEMICAL PROPERTIES**															
Bulk density (g/cm^3^)	^**^	^***^	^*^	^***^	^***^	^*^	^***^	^***^	ns	^***^	^***^	^**^	^***^	^***^	^**^
Porosity (%)	^*^	^***^	^**^	^*^	^***^	^*^	^*^	^***^	^***^	^**^	^***^	^**^	^**^	^***^	^**^
Soil moisture (%)	ns	^***^	^***^	ns	^***^	ns	^*^	^***^	ns	^*^	^***^	ns	^*^	^***^	ns
pH	^***^	^***^	^**^	^*^	^***^	ns	ns	^***^	ns	ns	^***^	ns	ns	^***^	ns
EC(μS/cm)	^***^	^***^	^***^	ns	^***^	^***^	ns	^***^	^***^	ns	^***^	^**^	ns	^***^	ns
**SOIL CARBON AND NUTRIENT IN CONCENTRATION**															
SOC concentration(g/kg)	ns	^***^	ns	ns	^***^	ns	ns	^***^	ns	ns	^***^	ns	ns	^***^	ns
Total N (g/kg)	ns	^***^	ns	ns	^***^	ns	ns	^***^	ns	ns	^***^	ns	ns	^***^	ns
Alkaline hydrolyzed N (mg/kg)	ns	^**^	ns	ns	^***^	ns	ns	^***^	ns	ns	^***^	ns	ns	^***^	ns
Total K (g/kg)	ns	ns	^**^	ns	^*^	^***^	^*^	^**^	^***^	^***^	^***^	^***^	ns	^***^	^***^
Available K(mg/kg)	^***^	^*^	ns	ns	^**^	ns	ns	^**^	ns	ns	^***^	ns	ns	^***^	ns
Total P (g/kg)	ns	^***^	ns	^*^	^***^	ns	^*^	^***^	ns	^*^	^***^	ns	ns	^***^	ns
Available P (mg/kg)	ns	^***^	^*^	ns	^***^	^*^	^*^	^***^	^**^	^*^	^***^	^**^	ns	^***^	^***^
**SOIL CARBON AND NUTRIENT IN STORAGE**															
SOC (kg/m^2^)	ns	^***^	ns	ns	^***^	ns	ns	^***^	ns	ns	^***^	ns	ns	^***^	ns
Total N (kg/m^2^)	ns	^***^	^*^	ns	^***^	ns	ns	^***^	ns	ns	^***^	^***^	ns	^***^	ns
Alkaline hydrolyzed N (g/m^2^)	ns	^**^	ns	ns	^***^	ns	ns	^***^	ns	ns	^***^	ns	ns	^***^	ns
Total K (kg/m^2^)	^**^	ns	^***^	^*^	^***^	^***^	ns	^***^	^***^	^*^	^***^	ns	ns	^***^	^***^
Available K(g/m^2^)	^***^	^**^	ns	ns	^**^	ns	ns	^***^	ns	ns	^***^	ns	ns	^***^	ns
Total P (kg/m^2^)	^*^	^***^	ns	^***^	^***^	ns	^***^	^***^	ns	^***^	^***^	ns	^*^	^***^	ns
Available P (g/m^2^)	ns	^***^	^*^	ns	^***^	ns	^**^	^***^	^*^	^**^	^***^	^**^	^*^	^***^	^***^

*** *p < 0.001*,

** *p < 0.01*,

* *p < 0.05, ns, no significant difference (p > 0.05) T, Type; L, Location*.

Compared with land use differences, there were even larger significant location-related differences among all parameters in all five soil depths. Moreover, significant interactions existed among the influence of land use and sampling location on some soil parameters in different layers (Table 1). For example, the influence of shelterbelt plantations on porosity, total K concentration, and available P concentration significantly interacted with location in the five depths, indicating that these shelterbelt-induced changes significantly differed among the six locations in all soil layers.

### Changes in Soil Properties Between Shelterbelt Plantations and Farmlands at Five Soil-Depth Profiles: Overall Patterns

The effects of shelterbelt construction on soil properties in the five soil-depth profiles and the differences in shelterbelt-induced soil changes in various parameters among the five profiles were shown in Table 2.

**Table 2:** A comparison in soil properties between shelterbelt plantation and farmland at five depths and the differences in shelterbelt-induced soil changes among five profiles.

	**Type**	**0–20 cm**	**0–40 cm**	**0–60 cm**	**0–80 cm**	**0–100 cm**
**PHYSIOCHEMICAL PROPERTIES**						
Bulk density (g/cm^3^)	Farmland	1.42	1.44	1.45	1.46	1.47
	Poplar	1.37^*^	1.38^***^	1.37^***^	1.40^***^	1.41^***^
	Change (%)	−3.1a	−4.1 a	−5.5 a	−4.2a	−4.3a
Porosity (%)	Farmland	42.30	41.13	39.99	39.03	38.87
	Poplar	45.33^*^	43.46^*^	42.08^*^	41.41^**^	40.76^**^
	Change (%)	13.7a	8.2a	8.0a	8.4a	6.7a
Soil moisture (%)	Farmland	12.56	13.41	13.16	12.67	12.35
	Poplar	12.92 ns	12.60^*^	12.19^**^	11.75^**^	11.42^***^
	Change (%)	6.2a	−7.3b	−8.7b	−8.0b	−7.9b
pH	Farmland	7.83	7.89	8.00	8.08	8.11
	Poplar	8.08^***^	8.04^***^	8.07 ns	8.14^*^	8.18^*^
	Change (%)	3.2a	2.0ab	0.9b	0.9b	0.9b
EC (μS/cm)	Farmland	159.85	127.78	116.87	112.16	108.39
	Poplar	105.22^***^	112.71 ns	113.07 ns	112.34 ns	108.45 ns
	Change (%)	−14.7b	−0.1ab	4.1a	6.2a	4.2a
**NUTRIENT CONCENTRATION**						
Total K (g/kg)	Farmland	44.38	48.28	47.17	48.36	50.84
	Poplar	40.34 ns	46.86 ns	49.26 ns	53.21 ^***^	52.55 ns
	Change (%)	27.6a	0.9a	7.3a	12.6a	5.5a
Available K (mg/kg)	Farmland	82.92	72.89	69.05	85.56	61.78
	Poplar	135.23^***^	89.34^***^	78.83^**^	99.16^***^	71.77^***^
	Change (%)	117.4a	39.2b	29.5b	26.3b	24.6b
Total P (g/kg)	Farmland	0.47	0.41	0.37	0.34	0.31
	Poplar	0.42 ns	0.37^*^	0.33^*^	0.31^*^	0.30 ns
	Change (%)	1.9a	−2.4a	−3.2a	−3.5a	0.6a
Available P (mg/kg)	Farmland	5.36	4.68	6.10	5.90	6.14
	Poplar	4.88 ns	4.15 ns	5.21 ns	5.16 ns	5.68 ns
	Change (%)	28.3a	6.1b	1.0b	−0.2b	3.5b
**NUTRIENT STORAGE**						
Total K (kg/m^2^)	Farmland	12.86	13.97	13.72	14.2	15.01
	Poplar	11.03^*^	12.97^*^	13.64 ns	14.92 ns	14.81 ns
	Change (%)	−0.7a	−2.8a	2.8a	8.1a	1.1a
Available K (g/m^2^)	Farmland	22.74	20.07	20.15	24.89	18.09
	Poplar	36.77^***^	24.46^**^	21.80 ns	27.53^*^	20.04^*^
	Change (%)	108.3a	33.6b	24.0b	21.1b	19.0b
Total P (kg/m^2^)	Farmland	0.13	0.12	0.11	0.10	0.09
	Poplar	0.11^*^	0.10^***^	0.09^**^	0.09^***^	0.08^*^
	Change (%)	−2.8a	−8.7a	−7.4a	−7.7a	−3.7a
Available P (g/m^2^)	Farmland	1.51	1.35	1.76	1.72	1.80
	Poplar	1.34 ns	1.14 ns	1.44^*^	1.44^*^	1.59 ns
	Change (%)	22.9a	2.1b	−3.0b	−4.3b	−0.5b

*** *indicates significant differences between shelterbelt plantation and farmland at different profiles at p < 0.001*,

** *indicates the significant differences at p < 0.01*,

* *indicates the significant differences at p < 0.05. ns indicates no significant difference (p > 0.05). The same letters denoted not significant difference among five profiles in shelterbelt-induced soil properties change (p > 0.05), while different letters denoted significant difference (p < 0.05). In addition, those parameters, which are not significant differences between shelterbelt plantation and farmland at different profiles and not significant difference among five profiles in shelterbelt-induced change among five profiles at the same time, are not shown in Table 2 (such as SOC, total N, and Alkaline hydrolyzed N)*.

The effects of shelterbelt construction on soil properties varied in five soil depth layers. Some indicators, such as bulk density, porosity, available K concentration, and total P storage, had significant difference at five soil depth. However, some indicators, such as SOC concentration (storage), total N concentration (storage), and available N concentration (storage) had no significant change on five profiles following shelterbelt establishment. Moreover, the effects of afforestation on the surface soils were more obvious than at depth for pH and EC. On the contrary, the effect of soil moisture was seen mainly in the deeper soil profiles (Table 2).

The significances among the five soil-depth profiles were distinct for different shelterbelt-induced soil properties changes. First, opposite trends (*p* < 0.05) were observed in shelterbelt-induced soil moisture and EC changes between surface and deeper soil layers. A 6.2% increase in soil moisture (poplar compared with farmland) was observed in the 0–20 cm layer, whereas there was a 7.3–8.7% decrease in the deep soil profiles. Contrary to soil moisture, EC was 14.7% lower in shelterbelt plantations than that in farmlands in the surface layer, but was 4.1, 6.2, and 4.2% higher in the 0–60, 0–80, 0–100 cm layers, respectively (Table 2). Second, the changes in available K and P were several-fold higher in the 0–20 cm soil profiles than that in the deeper profiles (*p* < 0.05). For example, a 117.4% increase in available K concentration was observed in the surface layer, whereas only a 24.6–39.2% increase was observed in the deeper layers. A 28.3% increase in available P concentration in shelterbelt plantations was observed in the surface layer, whereas a −0.2–6.1% change was found in the deeper layers. Third, no significant changes (*p* > 0.05) were found among the five soil profiles for the other properties.

### Shelterbelt-Induced Soil Parameter Changes: Large Inter-site Variations Differed With Soil Depth

**Figure 2:**
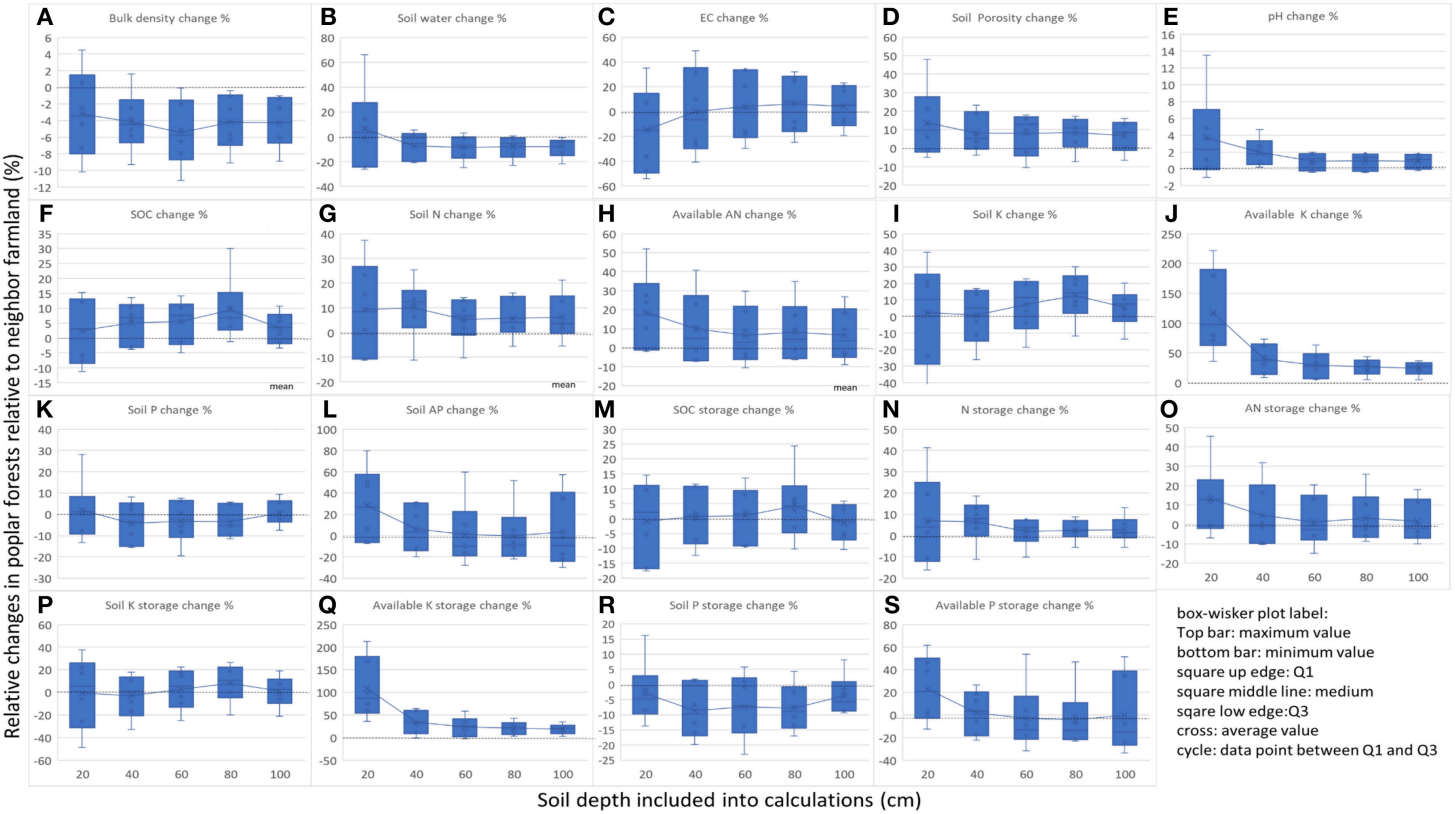
Changes of various soil properties in poplar forests compared with neighbor farmland, and differences at 1 m profiles. Dash line in the figure showed the zero line, indicating that no changes relative to neighbor farmland. Statistics of the mean values had shown in Table 2 and Table A1. **(A)** Soil bulk density change %; **(B)** Soil water change %; **(C)** Soil EC change %; **(D)** Soil porosity change %; **(E)** Soil pH change %; **(F)** Soil organic carbon concentration change %; **(G)** Soil total N concentration change %; **(H)** Soil alkaline hydrolyzed N concentration change %; **(I)** Soil total K concentration change %; **(J)** Soil alkaline K concentration change %; **(K)** Soil total P concentration change %; **(L)** Soil alkaline P concentration change %; **(M)** Soil organic carbon storage change %; **(N)** Soil total N storage change %; **(O)** Soil alkaline hydrolyzed N storage change %; **(P)** Soil total K storage change %; **(Q)** Soil alkaline K storage change %; **(R)** Soil total P storage change %; **(S)** Soil alkaline P storage change %.

All soil parameters except total K concentration and total K storage showed marked location-related differences in the five depths among the different sites (Table 1). The vertical pattern and magnitude of differences are shown in Figure 2 and Table A1.

The location differences were soil-depth dependent, i.e., in most cases, the surface soil layer showed much larger location differences in the poplar forest-induced changes in various soil properties (Figure 2). For example, inter-site differences in soil bulk density and soil water were −10 to 5% and −25 to 60% respectively, whereas those in deep soils (0–100 cm) were, respectively −9 to −1% and −20 to 0%. However, for other parameters, similar inter-site differences were found among surface and deep soils, with even larger variation in deep soils, for example, soil available P and SOC for both concentration and storage (Figure 2).

Depth-induced significant differences were observed in soil moisture, pH, EC, available K concentration and storage, and available P concentration and storage (Table 2), with large inter-site variations found among the different sites (Figure 2 and Table A1). There was a higher amount of available K concentration in the surface layer than that in the other four soil layers (Table 2). This trend mainly occurred in Dumeng, Fuyu, Mingshui, and Zhaodong (*p* < 0.05), with a 221.8% increase in the 0–20 cm layer and an average 0–20 cm layer and an average 46.8% increase in the other four profiles at Dumeng, whereas there was a 79.9% increase in the surface layer and an average 15.9% increase in the deeper profiles at Mingshui (Table A1). Moreover, although no significant differences were observed in three of the soil parameters (porosity, total K concentration, and total K storage) among the five soil layers (Table 2), inter-site differences were found among the different sites. For example, there were significant differences (*p* < 0.05) among the five soil profiles in total K concentration and storage at Dumeng, Fuyu, and Mingshui, with a 24.1% decrease in the 0–20 cm layer and an average 1.8% increase in the deeper profiles at Dumeng, and a 42.8% decrease in the surface and an average 17.5% decrease in the deeper profiles at Mingshui (Table A1).

### RDA Ordination: Climatic, Soil Texture and Forest Controls on the Inter-site Variations and Differences Between Surface and Deep Soils

As shown in Table 3, in general, climatic conditions provided the largest explaining power for the inter-site variations of shelterbelt-induced soil changes. Moreover, at different soil layers, MAT was the most influential parameter, providing the highest explanation percentage. For example, MAT explained 4.9, 5.1, 4.4, 3.0, and 3.5% of the forest-induced soil variations for 20, 40, 60, 80, and 100 cm depth soils, respectively (Table 3). In deeper soils, ARID and MAP explained much more of the variation than that in the surface soil layer. For example, ARID in 20 cm, 40 cm soils did not show significant explanation for the variations, while in 60, 80, and 100 cm soils, ARID showed significant explaining powers ranged from 3.0–4.6% (*p* < 0.05); and MAP explained 6.9% of the variations for 100 cm soil layers (*p* < 0.01) (Table 3).

**Table 3:** Comparison on the explaining power from climatic condition, soil texture, and forest characteristics for the forest-induced soil changes at different locations from the RDA ordination-related conditional term effects excluding their collinear effects.

**Soil inclusion**		**Explains %**	**pseudo-F**	***P***	**RDA ordination figure**
0–20 cm	Silt	7.8	5.9	0.002	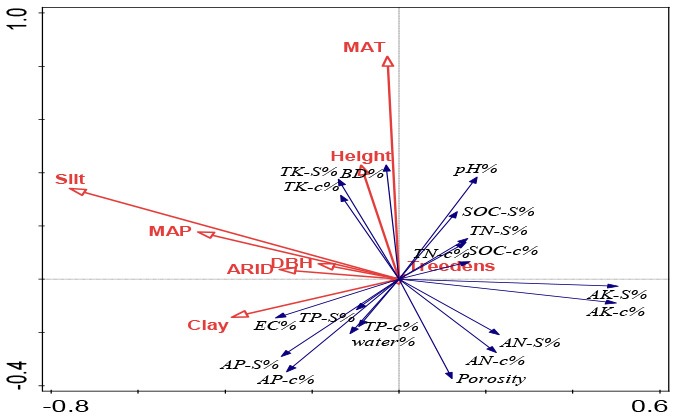
	MAT	4.9	3.9	0.004	
	DBH	3.1	2.6	0.028	
	MAP	2.8	2.2	0.042	
	ARID	1.4	1.2	0.276	
	Treedensity	1.3	1.1	0.374	
	Height	1.1	0.9	0.51	
0–40 cm	MAT	5.1	3.9	0.004	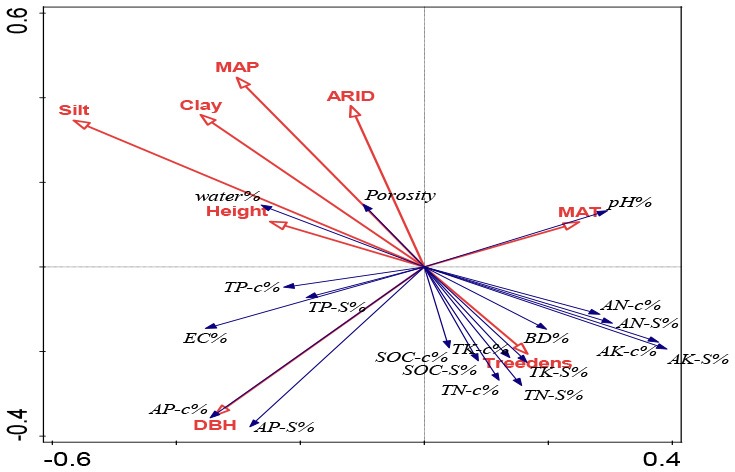
	Silt	4.5	3.3	0.004	
	Height	2.5	2	0.05	
	MAP	2.4	1.9	0.08	
	DBH	2.4	1.9	0.086	
	Treedensity	2	1.6	0.15	
	ARID	1	0.8	0.612	
	Clay	0.8	0.7	0.682	
0–60 cm	MAT	4.4	3.2	0.004	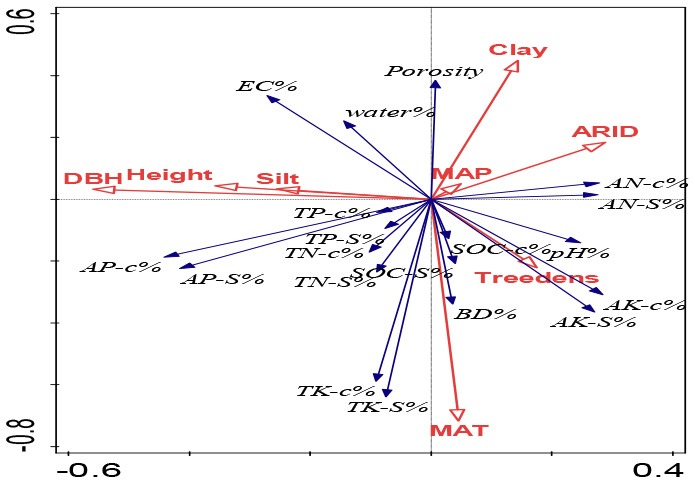
	DBH	3.6	2.8	0.008	
	Silt	3.3	2.5	0.01	
	ARID	3	2.2	0.036	
	Clay	1.8	1.4	0.182	
	Height	1.4	1.1	0.338	
	Treedensity	1	0.8	0.574	
	MAP	1	0.8	0.608	
0–80 cm	ARID	3.9	2.9	0.008	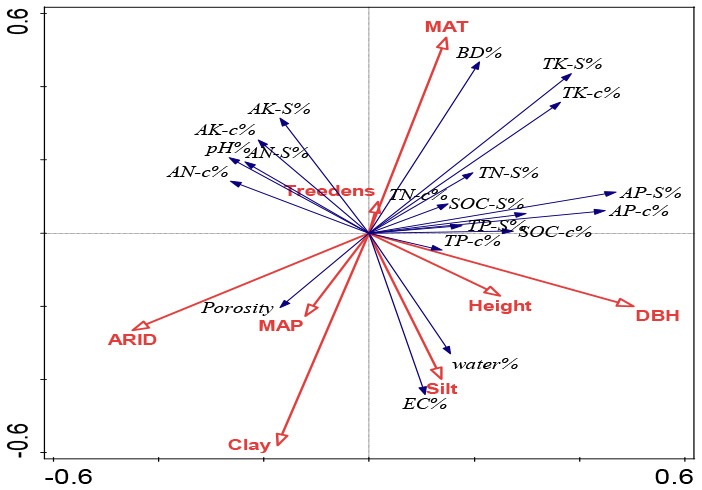
	DBH	3.5	2.7	0.01	
	Silt	3.5	2.7	0.018	
	MAT	3	2.1	0.036	
	Height	1.3	1	0.394	
	Treedensity	0.8	0.6	0.73	
	Clay	0.6	0.5	0.842	
	MAP	0.6	0.5	0.852	
0-100cm	MAP	6.9	5.3	0.002	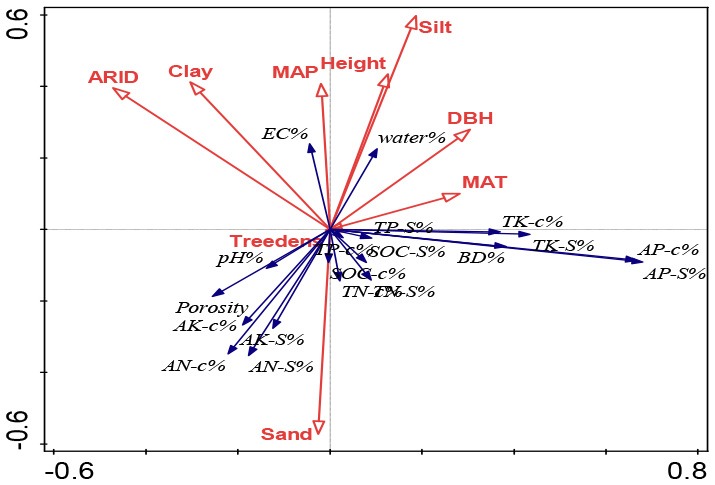
	ARID	4.6	3.4	0.006	
	MAT	3.5	2.8	0.01	
	DBH	2.7	2.2	0.034	
	Silt	2.1	1.7	0.124	
	Sand	1.5	1.2	0.276	
	Clay	1.4	1.2	0.308	
	Height	1.2	1	0.378	

Following the climatic conditions, soil texture gave the next largest explanation power for the location-related variations and silt showed significant explanation percentage in four out of five soil layers (*p* < 0.05) (Table 3). In general, the deep soils, the less explaining power from soil texture of silt percentage. In 20 and 40 cm soils, silt's explaining percentage was 4.5–7.8% (*p* < 0.001), and this percentage was 3.3–3.5% in 60 and 80 cm soils (*p* < 0.05), and no significant explaining percentage was found in 100 cm soil (*p* = 0.124) (Table 3).

In addition, tree growth traits (DBH and height) also significantly explained the shelterbelt-induced soil variations at different locations, and their explaining powers ranged from 2.5 to 3.6% at different soil layers (*p* < 0.05) (Table 3).

### Stepwise Regression Statistics: Factors Related to the Inter-site Variations and Differences Between Surface and Deep Soils

As a further step to decouple the association, stepwise regression analysis was used to determine the most possible parameters for the large inter-site variations (Table A2).

For each soil properties, we found different associations with climatic conditions. For example, positive correlations were observed between shelterbelt-induced soil bulk density change and MAT in all layers expect for the 0–40 cm depth. Although soil texture and tree growth significantly accounted for shelterbelt-induced porosity change in the 0–20 cm layer, MAT was the leading factor that determined the porosity change in the other four soil depths (*r*^2^ = 0.10–0.18, *p* < 0.05). The pH change was significantly related to MAT in the 0–20 and 0–40 cm depth layers, and the shelterbelt-induced EC decrease was accompanied with higher MAT in all five soil profiles. Significant positive correlations were found between SOC storage change, total K concentration (storage) changes, and MAT (*p* < 0.05). In the deep soil layers (0–80 and 0–100 cm), ARID was the significant affecting factor (*r*^2^ = 0.32, *p* < 0.001) for bulk density changes compared with the surface soil layer. Similar significant negative correlations were found between available P (concentration and storage) and ARID in the deeper soil profiles. Higher ARID accounted for the poplar-induced total K decrease in the five soil profiles (*r*^2^ = 0.15–0.46, *p* < 0.001), whereas the available K (concentration and storage) changes in the 0–100 cm depth could be explained by ARID and MAP (*r*^2^ = 0.39 and *r*^2^ = 0.40, respectively) (Table A2).

For soil silt percentage, significant positive correlations were found among silt percentage and EC, available P concentration, whereas marked negative correlations were found in the shelterbelt-induced differences in seven soil parameters in the different soil depths (including porosity, total N concentration, alkaline hydrolyzed N concentration, available K concentration, total N storage, alkaline hydrolyzed N storage, and available K storage) (Table A2). For tree growth parameters, DBH, Tree height, and Tree density have been found in different stepwise regression models in different soil layers; However, their appearances were not as often as that of climatic parameters and soil textures (Table A2).

**Figure 3:**
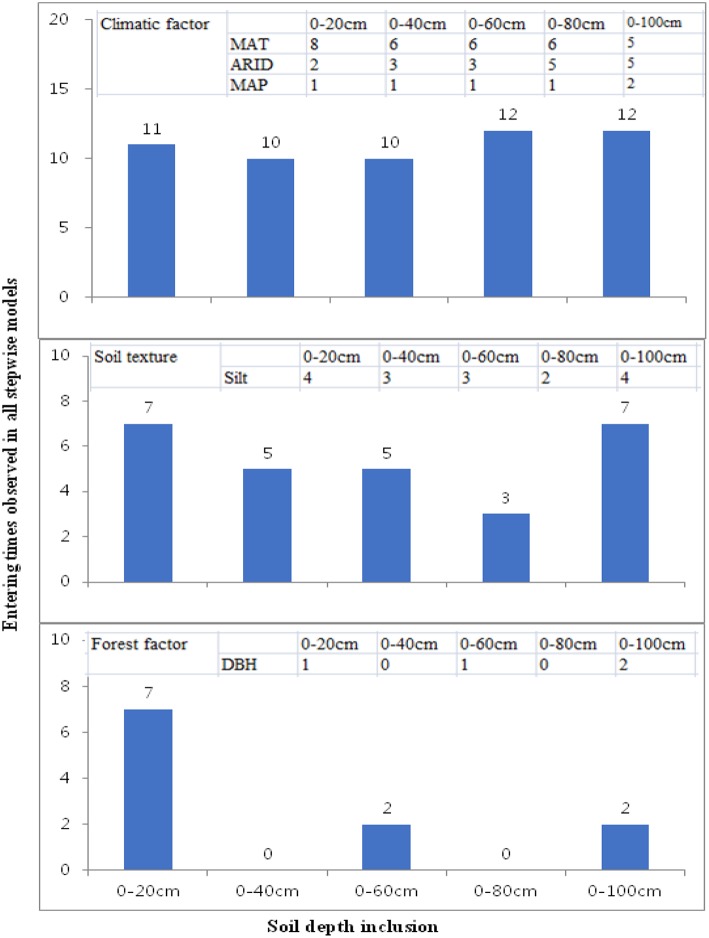
Differences of entering times of climatic factors (upper), soil texture (middle), and forest traits (lower) observed in the all stepwise regression models at different depths. Inset tables are the most observed parameters (MAT, ARID, MAP, Silt, and DBH) and their entering times all stepwise models at different soil depths. All the stepwise regression models were shown in Table A2. The more inclusion of the parameters into the stepwise models indicates their stronger contribution at that soil layers for explaining the forest-farmland differences in the studied soil properties.

By counting the entering times for each tested parameter observed in all stepwise regression models, we want to confirm the findings in RDA ordination, and the basic criteria is that the more entering times mean the more influences on soils from this parameter (Figure 3). Comparison among climate, soil texture and tree growth, we found the most entering times from climatic factors (10–12 entering times), followed by soil texture (3–7 entering times), and tree growth factors (0–7 entering times); This is the similar to those observed in RDA ordination (Table 3). In the case of different climatic parameters, we found that MAT showed the most influences (5–8 entering times), followed by ARID (2–5 entering times) and MAP (1–2 entering times) (Figure 3). At the vertical soil profiles, MAT's influences decreased from surface to deep soils, as shown by eight entering times in 20 cm soils and five entering times in 100 cm soils. However, ARID's influences showed a contrary pattern, i.e., lower influences were at surface soils (two entering times in 20 cm), while much stronger influences were in deep soils (five entering times) in 80 cm and 100 cm soils (Figure 3).

Soil texture showed the similar entering times at the surface (5–7 entering times) and deep (3–7 entering times) soils, and 2–4 times entering into stepwise models were observed from silt percentage at five depths soil (Figure 3). In the case of DBH, two entering times were found in 100 cm soil, while 0–1 time entering to the stepwise models was found in other soils (Figure 3).

## Discussion

### Higher Water Consumption in Deep Soils, Saline-Alkalinization in Surface Soils, and Better Physical Structure in the Entire 100 cm Soil Profile Following Shelterbelt Plantations

Our study highlighted significant water consumption in deeper soil layers in poplar forests in northeast China. There were significant decreases (7.3–8.7%) in 0–40 cm and deeper soils (*p* < 0.05), while a slight increase was found in 0–20 cm layer (Table 2). Artificial afforestation can decrease soil moisture because of leaf interception and root uptake (Jin et al., 2011). Divergent hydrological response to large-scale afforestation has been found on a national scale throughout China (Li Y. et al., 2018). Previous studies have shown that soil moisture differed significantly between traditional farmland and introduced woody vegetation (Wang et al., 2009a, 2011b; Liu et al., 2010; Li Y. et al., 2018). In north and southeast China, the increased precipitation and increased forest area were not statistically significant, and had only a weak influence on soil moisture content (Li Y. et al., 2018). In southwest China, however, the afforestation practices have been shown to significantly reduce soil moisture in combination with decreased precipitation (Li Y. et al., 2018). In northeastern China (the same region as in the present study), soil moisture has been shown to be significantly decreased by −8.1 mm decade^−1^ (Li Y. et al., 2018). High water consumption following afforestation has been reported as an important feature of fast-growth tree species afforestation (Yang et al., 2012; Jia et al., 2017; Liang et al., 2018). Songnen Plain (the central part of northeastern China) is characterized as experiencing land degradation with a shortage of precipitation (Li, 2000), and the high water consumption from poplar shelterbelt plantations possibly intensifies the degree of drought in the deeper soil depths, with an average precipitation of 400–500 mm and a large area of saline-alkalinization land (Zhang et al., 2013). Currently, measures used by local people to prevent this water consumption include digging a root-cutting ditch to hinder root invasion into farmland. Possible other measures to counteract the over-consumption of water by plantations forests have been proposed (Ferraz et al., 2013); for example, the selection of suitable tree species with low water utilization, such as *Picea* spp. (e.g., *P. jezoensis*), plays an important role in the reduction and regulation of water use (Wang et al., 2017a), and an increased proportion of native forests and mosaic management could also stabilize water flow across plantation landscapes.

Saline-alkalinization is an important feature of local land degradation in the Songnen Plain (Li, 2000) and different methods have been invented for soil improvement and afforestation practices in this region (Wang et al., 2011a). Our study found that, compared to farmland, the establishment of shelterbelts increased soil pH in the 0–20 cm and 0–40 cm soil layers (*p* < 0.001) (Table 2), whereas there were no significant differences in the deeper soil layers. Therefore, poplar afforestation resulted in surface soil saline-alkalinization in the Songnen Plain of China. Previous meta-analyses have found site-scale soil acidification globally (Berthrong et al., 2009) or afforestation-induced soil neutralizing pH that favored acidifying alkaline soils (Hong et al., 2018). Our findings were different from these meta-analyses, which may be related to the following. First, the addition of plant residues can increase, decrease, or have little effect on soil pH (Tang and Yu, 1999; Marschner and Noble, 2000; Xu et al., 2006; Rukshana et al., 2011), and are mainly dependent on the amount of returning organic materials. In poplar shelterbelt plantations, the relatively smaller area (i.e., several rows) could result in limited litter decomposition and rhizospheric processes following afforestation. Second, the increases in evapotranspiration (Yao et al., 2016) caused by poplar afforestation reduces the leaching loss of base cations (Slessarev et al., 2016), and thus increases soil pH. Third, the upward vertical movement of water by deep-rooted trees (compared with crops) could generally induce the upward movement of soluble salts from the deep soils to the surface soils, resulting in soil saline-alkalinization (Li, 2000; Lu et al., 2017; Wang et al., 2017a).

Planting shelterbelts with fast-growing species such as poplar causes soil bulk density to be significantly reduced in the 0–20 cm soil layer (Wu et al., 2018), and similar significant improvement in soil physics (e.g., bulk density decreasing and porosity increasing) were observed over the entire 100 cm soil profile including 0–40, 0–60, 0–80, and 0–100 cm soil layers (Tables 1, 2, *p* < 0.05). Soil physical structure is very important for soil function (Han et al., 2018) and previous studies have found surface soil improvements, for example, Marta and Halina (2008) observed that total porosity, on average, in the entire 20 cm horizon of the studied afforested soils was 1.08 and 1.12 times higher (for soils of young and older stands, respectively) than that in the arable soils. Our results are in agreeance with previous findings, emphasizing the importance of shelterbelt afforestation to deep soil layers. The long-term farmland cultivation in northeastern China has seriously degraded black soils, with one important aspect being the degradation of the soil physics (Li, 2000; Wang, 2002). Our results clearly show that shelterbelt afforestation could strongly improve soil physics and suggests a possible way for local soil improvement, such as returning degraded farmland to forests, with such policy being implemented in China over the past years (Wang et al., 2011b).

### Non-accrual of SOC Both in Surface and Deep Soil Layers Following Shelterbelt Forest Establishment

Results from various studies on the effects of afforestation on SOC are inconsistent. Some studies have found that afforestation increased SOC accumulation (Lemma et al., 2006; Wang et al., 2011b; Wei et al., 2012; Cukor et al., 2017), whereas other studies have shown that afforestation decreased SOC (Farley et al., 2004; Mao et al., 2010) or there was more initial loss than SOC gain (Paul et al., 2002; Wang et al., 2006; Ritter, 2007). In this paper, we did not find any significant changes between poplar shelterbelt plantations and neighboring farmlands.

According to our survey (data not shown here), 28 mg cm^−3^ poplar root system was in 1 m depth, and 95% of the root was distributed in 0–60 cm soil layer, especially in 20–40 cm (57%). Previous studies have also highlighted the possible differences in different soil layers, owing to the root differences between trees (long roots) and crops (short roots) (Wang H. M. et al., 2014). For example, Hooker and Compton (2003) found that SOC linearly accumulated in the subsoil (20–70 cm), but did not differ in the top 20 cm after afforestation. Wang H. M. et al. (2014) reported that, in larch forest plantations, the rate of change in SOM in the surface soil was 262.1 g kg^−1^year^−1^; however, a different trend in deeper soils resulted in no evident changes in the overall 80 cm soil profile. Our previous paper found no shelterbelt-induced SOC accumulation in the 0–20 cm soil layer (Wu et al., 2018). In the present study, we confirmed a similar finding (i.e., no significant SOC changes) in the 0–40, 0–60, 0–80, and 0–100 cm soil layers (Tables 1, 2, Table A1).

Possible reasons for the above-mentioned patterns include the following. First, the shelterbelt poplar planting area was generally 4–6 rows of poplars around large farmlands of ~25 ha in size (Figure 1). In this type of shelterbelt forest, the canopy litter is usually deposited on both farmland and forest simultaneously, which reduces the influence of shelterbelt poplar to forest soils with reference to neighboring farmlands. Second, high productive crops and tillage practices diminish the differences between farmland and forests. It is generally assumed that forests can improve soil carbon sequestration; however, different crops and tillage practices change this sequestration. In the present study, the high productive C4 crop (maize) was the main crop in this region and soybean was the second largest crop with high N-fixation ability (this N fixation favors SOC sequestration) (Lian et al., 2017; You et al., 2017). Proper chemical fertilizer utilization together with straw returning, which has been strongly implemented by the local government, improves the stabilization and accumulation of SOC (Li and Han, 2016). No chemical fertilization or organic manure were applied in the management of the shelterbelt forest, which was different from the neighboring farmlands. Third, microbial priming-induced SOC loss possibly also contributed to the patterns (Li L. J. et al., 2018). The root-exudate inputs in the deep soil could stimulate the decomposition of SOC by priming soil microbial activity (Marie-Anne et al., 2014), and the SOC mineralization might stimulate loss of the deeper SOC pool (Fontaine et al., 2007), which possibly resulted in the non-accrual of SOC storage in the deeper profiles in the poplar shelterbelts.

### Non-evident Changes in all Nutrients Except Available K Recovery in 5 Soil Layers and Total P Depletion in Deep Soils Following Shelterbelt Afforestation

Shelterbelt-induced available K recovery was found in all five soil depths (*p* < 0.05) (Table 2), showing that K accumulation was not only in the surface soils but was also in the deeper soils. Returning farmland to forest could rehabilitate the soil K fertility in different areas worldwide using different tree species (Likens et al., 1994; Romanowicz et al., 1996). In China, Jiao et al. (2012) observed that available K was significantly higher in afforested sites than that in degraded croplands in the Loess Plateau. In the case of farmland fertilization practices, more K fertilizer together with N (the favorite fertilizer of local people) should be applied to ensure soil nutrient supply for crop productivity.

In the present study, shelterbelt-induced total P depletion was observed mainly in the deeper soil layers (*p* < 0.05) (Table 2). In large areas of larch plantations in northeastern China, Wang W. J. et al. (2014) observed that more P was stored in deeper soil layers, and >70% of P (total and extractable) was found in deeper soil layers (20–80 cm) during larch reforestation. The development of larch plantations could result in a general uplifting of SOM, N, and P based on vertical distribution data and this redistribution was accompanied by the depletion of N and P. The soil nutrient depletion could be related to the biological uplifting function and possible absorption related to tree growth (Jobbágy and Jackson, 2004). In addition, the differences in microbial decomposability between deep and surface soils might strengthen the depletion in deep soil layers (Fontaine et al., 2007; Xiang et al., 2008).

All tested nutrients including N, available N, available P, and total K did not significantly change throughout the entire 100 cm soil profile in shelterbelt poplar forests with reference to neighboring farmlands in a large-scale field in this paper (Table 2). A general observation from field surveys is that the growth of crops near the shelterbelt poplar is smaller and general assumptions are that shelterbelt afforestation can decrease soil nutrients owing to nutrient competition between poplar and crops. By the entire soil profile measurements in the present study, we updated this assumption and found that the most likely nutrient depletion was that of P depletion. However, for almost all other nutrients, such depletion was not found either in surface soils or deeper soils. This should be taken into consideration in future shelterbelt forest evaluations.

### Large Inter-site Variations Closely Associated With Climatic Conditions and Deep Soils Showed Greater Dependence on Arid and Map: Implications

To determine the differences between plantation forest and neighboring farmlands, many previous studies have looked at a relatively smaller region to minimize the inferences of inter-site variation on the forest effects (Mao et al., 2010; Wang et al., 2011b; Wei et al., 2012; Cukor et al., 2017). In the present study, large-scale sampling was undertaken in Songnen Plain (at least 33,000 km^2^) to determine the general soil change patterns in a 100 cm soil profile. Large inter-site variations in the shelterbelt-induced soil changes were found (Figure 2 and Table A1). Currently, global climatic changes strongly affect local development including natural processes in farmlands, pastures, and forests (Li, 2000; Li Y. et al., 2018) and new developments has been reported in tree inventory methods (Wang et al., 2018a) and complex association analysis (Lv et al., 2018; Wang et al., 2018b; Yang et al., 2019). Decoupling the contribution of different components on inter-site variations of the shelterbelt-induced soil changes will favor the mechanical understanding of underlying processes, and is a possible strategy for large scale evaluation of shelterbelt poplar ecological functions (Wang et al., 2018b; Yang et al., 2019).

To identify the possible contributions from climatic conditions, soil texture, and tree growth that affected soil property changes following afforestation, statistical methods including RDA and stepwise regression analysis were applied, which have been proven as beneficial for determining the causal relationship between patterns (Eisenhauer et al., 2015; Wang et al., 2018b; Yang et al., 2019). Accordingly, we found that the inter-site variations in the shelterbelt-induced soil changes in surface soils were different from those in deeper soils.

In general, MAT was the most important parameter with the highest explanation powers, and changes in seven parameters (bulk density, porosity, soil moisture, pH, EC, SOC stock, and total K in different soil layers) were significantly accounted for by the variations in MAT (Figure 3, Table 3, Table A2). This is related to the fact that temperature is one of the main factors limiting soil nutrients following the planting of plantations (Wiesmeier et al., 2013). Similar to our conclusion, climate warming has been strongly associated with a decrease in the accumulation of glomalin proportion to total soil carbon in soils (Wang et al., 2017b). Moreover, RDA and stepwise regression analysis indicated that ARID and MAP gave greater explanation percentages in deeper soils than those in surface soils (Figure 3, Table 3, Table A2).

Potential evapotranspiration (PE) and aridity index (ARID) based on the observational data from 1961 to 2004 from 94 meteorological stations showed a general increasing trend in MAT, MAP, PE, and ARID (Zhao et al., 2007). Moreover, increases were more significant in MAT and PE than that in MAP and ARID (Zhao et al., 2007), showing that northeastern China is the most serious region experiencing global changes, especially for temperature warming. The ARID range of the six locations in Songnen plain is 0.49–0.67 and the semi-arid climate possibly affects the shelter-induced soil properties changes. Our findings indicate that global changes will shift the shelterbelt-induced soil changes with reference to neighboring farmlands. Moreover, different changes have been found between surface and deep soils. Compared with surface soils, the drying trends will give more influences in deep soils owing to the large explanation powers compared with the surface soils (Figure 3, Table 3, Table A2). The Three-North Shelterbelt Program has been evaluated as the most important natural environmental rehabilitation program in China (Bryan et al., 2018), and our findings highlight that soil changes should be carefully considered during any evaluation and divergent responses to climatic changes should be included in risk assessments.

## Conclusion

By analyzing 720 soil samples from 72 paired sites of poplar shelterbelts and farmlands in Songnen Plain in northeastern China, we concluded the following: (1) Shelterbelt poplar plantations significantly improved soil physical properties by decreasing bulk density and increasing porosity down into the 100 cm depth; however, higher water consumption was mainly found in the deep soils and soil saline-alkalinization was mainly in the surface soils. (2) There were no evident changes in all nutrients except for available K recovery following shelterbelt afforestation in all five soil depths and shelterbelt-induced total P depletion occurred mainly in deep soils. (3) Large inter-site variations were found for all shelterbelt-induced soil changes (*p* < 0.05) except for total K in the 0–20 cm soil layer, and MAT and soil texture were the largest explanation powers for soil property changes in the different soil layers. However, in deeper soils, soil drought (ARID and MAP) gave more explanation percentages than that in surface soils. Our findings highlight that shelterbelt poplar plantations could divergently change different soil properties in different soil depths, and inter-site variation was strongly associated with climatic changes. Our findings favor shelterbelt poplar forest evaluation and the underlying reasons for the large inter-site variation could help find suitable parameters to reduce the uncertainty of future evaluations.

## Author Contributions

WW, SH, YW, QW, and HW conceived and designed the experiments. WW and YW contributed reagents, materials, and analysis tools. QW, YW performed the experiments. WW, SH, QW, and YW analyzed the data. WW and YW wrote the paper. All authors approved the submitted and final versions.

### Conflict of Interest Statement

The authors declare that the research was conducted in the absence of any commercial or financial relationships that could be construed as a potential conflict of interest.
