# Supplementary material for: Shelterbelt Poplar Forests Induced Soil Changes in Deep Soil Profiles and Climates Contributed Their Inter-site Variations in Dryland Regions, Northeastern China
Source: Front Plant Sci. 2019 Mar 5;10:220. doi: 10.3389/fpls.2019.00220 (PMC6416468; doi:10.3389/fpls.2019.00220)
Supplement: Supplementary file 1 [file Data_Sheet_1.pdf]

Table A1 A comparison in soil properties change among locations at five depths

Location	Depth	Relative changes in physiochemical properties %					Relative changes in soil carbon and nutrients in concentration %							Relative changes in soil carbon and nutrients in storage %						
	(cm)	BD	PR	SM	pH	EC	SOC	TN	AN	TK	AK	TP	AP	SOS	TNS	ANS	TKS	AKS	TPS	APS
Dumeng	0-20	-2.5 a	13.3 a	-24.0a	3.6a	-48.0b	12.1a	23.3a	52.0a	-24.1b	221.8a	1.7a	6.4a	9.9a	19.5a	45.5a	-25.8 b	212.2a	-1.5a	2.8a
	0-40	-5.1 a	6.8 a	-20.6a	2.9a	-26.4ab	7.6a	25.4a	40.8a	-3.3ab	73.5b	-15.5a	19.0a	1.8a	18.5 a	31.9a	-8.4ab	64.6b	-19.8 a	12.6a
	0-60	-6.5 a	16.8 a	-24.9a	1.8a	-15.6a	7.5a	14.1a	29.7a	3.4a	44.4b	-8.1a	10.5a	1.0a	7.4 a	20.3a	-3.7a	34.6b	-13.4 a	4.2a
	0-80	-5.7 a	17.1 a	-23.0a	1.8a	-8.4a	10.3a	16.0a	34.8a	6.5 a	36.5b	-11.4a	5.3a	2.9a	8.8 a	25.8a	0.1a	29.3b	-17.0 a	-0.9a
	0-100	-5.9 a	16.1 a	-21.6a	1.7a	-6.3a	10.7a	21.2a	26.7a	0.5a	32.8b	-2.3a	-1.8a	3.1 a	13.2 a	17.9a	-6.0ab	25.0b	-8.6 a	-7.7a
Fuyu	0-20	-7.3a	48.0a	14.5a	1.1a	7.0 a	-11.2a	15.5a	23.9a	2.4ab	115.8a	28.0a	50.5a	-17.5 a	6.4 a	14.1a	-5.9 ab	98.9 a	16.2 a	38.9a
	0-40	-5.8a	23.3b	5.6b	0.6 ab	31.1 a	6.1a	13.4a	22.9a	-11.2b	44.1b	8.2a	-12.4ab	-0.3 a	6.2 a	16.5a	-16.7 b	36.3b	1.2 a	-17.3 ab
	0-60	-5.1a	15.8b	3.1b	-0.2 ab	33.5a	7.7a	13.0a	19.1a	-3.9ab	22.4b	6.2a	-27.9b	2.2 a	7.3 a	13.4 a	-9.2 ab	15.0b	1.1 a	-31.6 b
	0-80	-6.3a	10.9b	0.8b	-0.4 b	32.0 a	4.0 a	14.1a	17.1a	9.1a	23.1b	5.0a	-18.5ab	-3.0a	6.5 a	10.3 a	2.1a	15.5 b	-2.4 a	-23.2 ab
	0-100	-6.0a	8.6b	-0.8b	0.0 ab	22.9 a	1.4a	12.8a	18.3a	4.6ab	17.9b	5.5a	-29.7 b	-5.1 a	5.6 a	11.6 a	-1.9 ab	10.7 b	-1.4 a	-33.6 b
Lanling	0-20	4.5a	-1.3a	-26.0a	13.5a	-36.4b	12.4a	37.4a	10.2a	18.6a	71.2a	-7.9a	-6.1a	14.6a	41.3a	11.7 a	22.1a	74.6a	-7.9 a	-0.1a
	0-40	1.6a	0.6a	-19.9a	4.7b	-23.1ab	10.4a	11.3a	-1.4a	15.5a	31.5a	-15.1a	-11.9a	11.6a	13.0a	-1.3a	17.9a	31.0a	-16.0a	-5.6 a
	0-60	-0.1a	-2.2a	-14.7a	2.0b	-18.1ab	14.2a	4.3a	-4.9a	22.7a	34.4a	0.6 a	-9.4a	13.6 a	3.4a	-5.6 a	22.3a	35.4a	-0.8a	-8.8 a
	0-80	-0.4a	3.1a	-14.4a	1.8b	-13.3 a	6.9a	1.9a	-5.7a	19.4a	31.8a	-9.9a	-21.7 a	6.5a	1.8a	-6.1a	18.9a	29.9a	-10.6 a	-21.3 a
	0-100	-1.3a	0.5a	-13.1a	0.9b	-9.0 a	7.1a	1.4a	-8.9a	10.8a	23.9 a	-1.3a	-22.3a	5.8a	0.4a	-10.1a	9.3 a	21.2 a	-2.8a	-22.9 a
Mingshui	0-20	-10.2a	21.0a	66.0a	-1.0a	7.8a	-7.7a	-10.7a	27.6a	-42.8b	79.9a	0.8a	79.7a	-16.5 a	-10.7a	15.4a	-48.7 b	59.3a	-8.5a	61.8a
	0-40	-9.3a	18.5a	1.6b	0.9a	9.6a	-3.8a	-11.2a	10.7a	-26.0a	8.5b	2.4a	30.5b	-12.4a	-11.2a	0.9 a	-32.8 a	-1.3 b	-6.7 a	18.6b
	0-60	-7.9a	17.9a	-0.9b	0.6 a	20.2 a	-1.3a	-10.2a	9.2a	-18.5a	6.1b	-5.7a	-10.3c	-9.4a	-10.2 a	0.2 a	-24.9 a	-3.0 b	-13.7 a	-17.4c
	0-80	-9.1a	15.1a	-1.1b	1.3 a	27.4a	-1.2a	-5.6 a	9.7a	-11.9a	17.8b	-5.1a	-11.9c	-10.2 a	-5.6 a	-0.2 a	-19.9 a	7.3 b	-13.6a	-20.0 c
	0-100	-8.9a	13.3a	-3.5b	1.8a	20.5a	-1.5a	-5.5 a	9.7a	-13.6a	31.3b	0.1a	-17.3c	-10.4a	-5.5 a	-0.1a	-21.2 a	19.7 b	-8.6a	-24.7 c
Zhaodong	0-20	-4.4a	5.9a	7.6a	4.9a	-54.0 b	15.3a	-11.2a	-2.0a	21.1a	179.7a	1.9a	-7.6b	9.6a	-16.2a	-6.9a	16.8a	168.7a	-1.5a	-12.5 b

	0-40	-2.5a	-3.7 a	-4.3ab	2.7b	-40.6ab	13.6a	6.3a	-7.2a	13.5a	62.8b	4.3a	-20.0b	10.7a	3.4a	-9.7a	10.8 a	59.4b	1.8a	-22.2 b
	0-60	-2.0a	-10.4a	-7.1b	1.8bc	-29.5 ab	10.4a	1.9a	-3.4a	20.7a	62.9b	7.6a	-16.3b	8.0 a	-0.3a	-5.8a	17.9a	58.5b	5.8a	-18.3 b
	0-80	-1.1a	-7.3a	-4.4ab	1.5c	-25.0a	6.7a	2.5a	-0.8a	22.6a	43.8b	5.8a	-6.4b	5.3a	1.1a	-2.3 a	21.1 a	42.1b	4.4a	-7.1 b
	0-100	-1.0 a	-6.6a	-3.6ab	1.3c	-19.2 a	5.6a	2.9a	-2.4 a	20.2a	36.5b	9.3a	35.1a	4.4a	1.6 a	-3.7 a	18.8a	35.0b	8.1a	34.6 a
Zhaozhou	0-20	0.5a	-4.9a	-0.7a	0.2a	35.1a	-5.9a	1.2a	-1.2a	38.9a	36.1a	-13.3a	47.0a	-5.4a	1.3a	-0.5 a	37.6a	36.0a	-13.8 a	46.6 a
	0-40	-3.8a	3.6a	-6.4a	0.2a	48.7 a	-3.0a	14.4a	-6.8a	16.8a	15.1a	-9.3a	31.3a	-7.2 a	9.8a	-10.2 a	12.4a	11.5 a	-12.8 a	26.3 a
	0-60	-11.2a	10.0a	-7.6a	-0.4 a	33.8 a	-5.0a	9.1a	-10.5a	19.5a	6.6a	-19.6 a	59.7a	-9.2a	4.2 a	-14.8a	14.5a	3.2 a	-23.1 a	53.7 a
	0-80	-2.6a	11.1a	-6.1a	-0.3 a	24.3a	30.1a	5.6a	-6.3a	30.0a	4.9a	-5.4a	51.7a	24.3 a	2.6 a	-8.8 a	26.5 a	2.5 a	-7.2 a	46.8 a
	0-100	-2.5a	8.4a	-4.4a	-0.2 a	16.4 a	-3.3a	4.1a	-3.9a	10.4a	5.1a	-7.5a	57.2a	-6.1 a	1.2 a	-6.3 a	7.5 a	2.4 a	-9.3 a	51.6 a

Table A2 Stepwise regression analysis between soil properties change and tree growth, soil texture and climate factors at different depths

Dependent variable	0-20cm	0-40cm	0-60cm	0-80cm	0-100cm
Soil physiochemical properties					
BD change	=-29.3+7.5* MAT, r^2=0.15		=-20.8+4.8* MAT, r^2=0.15	=-16.1+6.4* MAT-18.3*ARID, r^2=0.32	=-13.5+5.9* MAT-19.9*ARID, r^2=0.34
PR Change	=55.3+2.4*Clay-0.9*Silt-2.1*TH -0.4*TD, r^2=0.29	=-57.2-14.2* MAT, r^2=0.11	=66.5-17.0* MAT, r^2=0.18	=46.5-11.1* MAT, r^2=0.10	=44.7-11.0* MAT, r^2=0.12
SM change	=60.6+3.3* Clay-33.7*MAT, r^2=0.18	=14.1+1.8* Clay-102.6*ARID, r^2=0.18	=-30.1+1.0*Clay, r^2=0.09	=36.0-0.4*Sand-8.8*MAT, r^2=0.11	=-27.4+0.8*Clay, r^2=0.08
pH change	=-24.1+8.1* MAT, r^2=0.23	=-5.5+2.1*MAT, r^2=0.06			
EC change	=76.6-41.0* MAT+1.2*Silt, r^2=0.15	=91.1-26.4* MAT, r^2=0.04	=101.0-28.1* MAT, r^2=0.10	=99.2-35.7* MAT+0.7*Silt, r^2=0.17	=74.8-20.4* MAT, r^2=0.09
Soil carbon and nutrients in concentrations					
TN change		=193.4-0.4*MAP, r^2=0.04	=148.8-0.3*MAP, r^2=0.04		=33.4-0.6*Silt, r^2=0.07
AN change	=111.7-4.2*TH-20.8*TD, r^2=0.15	=65.7-1.3*Silt, r^2=0.10	=53.6-1.1*Silt, r^2=0.08	=149.9-0.3*MAP, r^2=0.04	=47.3-0.9*Silt, r^2=0.08
TK change	=-19.8+46.8* MAT-252.2*ARID, r^2=0.15	=-33.3+32.1*MAT-138.3*ARID, r^2=0.27	=-32.3+31.1*MAT-122.1*ARID, r^2=0.38	=7.6+24.8*MAT-144.9*ARID, r^2=0.29	=-6.8+19.2*MAT-97.5*ARID, r^2=0.22
AK change	=-893.3-7.7*Silt+2.9*MAP, r^2=0.20	=96.3-1.3*Silt, r^2=0.08			=-586.9-727.5*ARID+2.2*MAP +1.0*DBH-2.3*Clay, r^2=0.39
TP change	=41.9-2.7*TH, r^2=0.07				
AP change	=184.5-45.3* MAT, r^2=0.07		=120.5-361.9*ARID+1.2*DBH +1.2*Silt, r^2=0.15	=129.5-233.8*ARID, r^2=0.12	

Soil carbon and nutrients in storage					
SOS change	=-64.5+18.4*MAT, r^2=0.08	=-42.5+12.5*MAT, r^2=0.04			
TNS change					=24.4-0.5*Silt, r^2=0.06
AN S change	=-18.7+0.8*Sand, r^2=0.07	=55.3-1.2*Silt, r^2=0.10	=40.2-0.9*Silt, r^2=0.06	=48.4-1.0*Silt, r^2=0.10	=37.3-0.8*Silt, r^2=0.07
T KS change	=-50.3+53.9*MAT-245.6*ARID, r^2=0.19	=-57.9+37.4*MAT-133.0*ARID, r^2=0.32	=-52.7+34.8*MAT-116.5*ARID, r^2=0.46	=-11.5+30.4*MAT-154.0*ARID, r^2=0.39	=-21.8+24.3*MAT-109.9*ARID, r^2=0.33
AKS change	=263.5-3.6*Silt, r^2=0.16	=-1.11+0.9*Sand, r^2=0.07	=-51.4+33.7*MAT-2.8*TH, r^2=0.11		=-615.0-728.6*ARID+2.3*MAP -2.4*Clay+0.9*DBH, r^2=0.40
APS change	=6.3+3.0*DBH-5.0*TH, r^2=0.15		=45.0-2.2*Clay, r^2=0.06	=118.9-221.9*ARID, r^2=0.11	
Data statistics					
Climatic entering times	11	10	10	12	12
Soil texture entering times	7	5	5	3	7
Forest parameter entering	7	0	2	0	2

Note: The abbreviation in Table A1 and A2: BD: Bulk density; PC :Porosity; SM: Soil moisture; TN: Total N; AN: Alkaline hydrolyzed N; TK: Total K; AK: Available K; AP: Available P; SOS:Soil organic carbon storage; TNS: Total N storage; ANS: Alkaline hydrolyzed N storage; TKS: Total K storage; AKS: Available K storage; TPS: Total P storage; APS: Available P storage.
